# Neuroserpin and Extracellular Vesicles in Ischemic Stroke: Partners in Neuroprotection?

**DOI:** 10.14336/AD.2024.0518

**Published:** 2024-10-01

**Authors:** Santra Brenna, Markus Glatzel, Tim Magnus, Berta Puig, Giovanna Galliciotti

**Affiliations:** ^1^Experimental Research in Stroke and Inflammation (ERSI) Group, Department of Neurology, University Medical Center Hamburg-Eppendorf, 20246 Hamburg, Germany.; ^2^Institute of Neuropathology, University Medical Center Hamburg-Eppendorf, 20246 Hamburg, Germany

**Keywords:** ischemic stroke, tPA, neuroserpin, extracellular vesicles, blood-brain barrier, microglia

## Abstract

Ischemic stroke represents a significant global health challenge, often resulting in death or long-term disability, particularly among the elderly, where advancing age stands as the most unmodifiable risk factor. Arising from the blockage of a brain-feeding artery, the only therapies available to date aim at removing the blood clot to restore cerebral blood flow and rescue neuronal cells from death. The prevailing treatment approach involves thrombolysis by administration of recombinant tissue plasminogen activator (tPA), albeit with a critical time constraint. Timely intervention is imperative, given that delayed thrombolysis increases tPA leakage into the brain parenchyma, causing harmful effects. Strategies to preserve tPA's vascular benefits while shielding brain cells from its toxicity have been explored. Notably, administering neuroserpin (Ns), a brain-specific tPA inhibitor, represents one such approach. Following ischemic stroke, Ns levels rise and correlate with favorable post-stroke outcomes. Studies in rodent models of focal cerebral ischemia have demonstrated the beneficial effects of Ns administration. Ns treatment maintains blood-brain barrier (BBB) integrity, reducing stroke volume. Conversely, Ns-deficient animals exhibit larger stroke injury, increased BBB permeability and enhanced microglia activation. Furthermore, Ns administration extends the therapeutic window for tPA intervention, underscoring its potential in stroke management. Remarkably, our investigation reveals the presence of Ns within extracellular vesicles (EVs), small membrane-surrounded particles released by all cells and critical for intercellular communication. EVs influence disease outcome following stroke through cargo transfer between cells. Clarifying the role of EVs containing NS could open up urgently needed novel therapeutic approaches to improve post-ischemic stroke outcome.

## Ischemic stroke, a complex condition with many players

Ischemic stroke arises from decreased blood supply to a portion of the brain, leading to insufficient oxygen and nutrient delivery, thereby causing significant dysfunction of the affected tissue. Typically triggered by a thrombus or embolus blocking a cerebral blood vessel, this condition sets off a cascade of complex pathophysiological responses. At the ischemic core, cells undergo rapid death through necrosis. In the surrounding 'penumbra' area, cells are sustained by residual blood flow and are maintained metabolically active but electrically silent, and depending on various factors they may either undergo apoptosis or survive [1, 2].

Apart from neuronal loss, a prominent feature in stroke pathogenesis is the disruption of the blood-brain barrier (BBB). This occurs due to activation of matrix metalloproteinases which degrade the basal lamina and tight junctions of endothelial cells. Consequently, immune cells from the bloodstream, including macrophages, neutrophils, lymphocytes and dendritic cells infiltrate the brain, exacerbating the inflammation at initial stages, but also supporting recovery at later time points [3]. Furthermore, dying cells release damage-associated molecular patterns (DAMPs), triggering inflammation and promptly activating microglia, which proliferate and migrate to the lesion site [4]. As with the infiltrating cells, microglia also play a double role. In the acute phase of stroke, microglia contribute to ischemic damage by releasing pro-inflammatory cytokines such as TNFα, IL-1β, C1q or IL-6 [5]. Following this phase, they transition to a beneficial role, releasing critical neuroprotective factors and promoting tissue regeneration and angiogenesis [6-8]. A similar pattern is observed for astrocytes. The cytokines released by microglia activate astrocytes, which can intensify neuronal death by releasing inflammatory mediators and free radicals [9, 10] but at a later time point they can transition to playing a protective role by facilitating the formation of a glial scar, which limits inflammation and shields the surrounding healthy tissue [11-13].

Despite stroke being recognized by the World Health Organization as one of the main causes of mortality and disability worldwide, with ischemic stroke accounting for 87% of the cases and hemorrhagic stroke for the remaining, the available therapeutic options remain limited [14, 15]. Currently, the delivery of recombinant tissue plasminogen activator (tPA) and mechanical thrombectomy are the sole approved treatments. These interventions aim to promptly reperfuse the affected area, but they are recommended within a narrow time window of 4.5 to 6 hours following the onset of stroke symptoms. Although recent studies indicate that in a few cases perfusion imaging techniques such as computed tomography or magnetic resonance imaging might be used to expand this time window [16-18], strict inclusion criteria limit the eligibility of many patients. Moreover, the scarcity of resources and expertise, particularly in smaller medical facilities, restricts access to thrombectomy. Consequently, a significant portion of stroke patients are unable to benefit from these interventions. Hence, there is a pressing need for novel therapeutic approaches [19-21]. In the present review, we focus on the concept that a deeper understanding of the mechanisms of action of tPA and its inhibitors may indeed unlock avenues for new promising treatments.

### tPA beyond thrombolysis

tPA, initially identified in the 1940s as a fibrinolytic substance by a Danish research group [22], underwent significant advancements over subsequent decades. Particularly in the 1970s, researchers achieved a breakthrough by purifying tPA from melanoma cells [23]. This progress culminated in the approval of thrombolytic therapy in the 1990s. This treatment, based on intravenous administration of recombinant tPA, was designed to mitigate the adverse effects of ischemic stroke and enhance clinical outcomes [24]. However, the treatment was initially recommended within three hours of symptom onset, as tPA administration beyond this therapeutic time window increased the risk of hemorrhages and aggravated neuronal death in the parenchyma. Indeed, in the same years, tPA’s role in promoting neuronal death began to be elucidated. Studies on knock-out mice lacking the serine protease revealed that the absence of tPA protected neurons from excitotoxic-induced death, suggesting that tPA might mediate neuronal damage [25]. Given that excitotoxicity is one mechanism mediating neuronal injury after cerebral infarct [26, 27], to clarify tPA’s role in the post-stroke aftermath investigations were conducted in mice subjected to focal cerebral ischemia following middle cerebral artery occlusion (MCAO) [28]. Once again, the absence of tPA turned out to be protective, as tPA-deficient mice showed 50% smaller infarcts, increased neuronal survival and improved long-term outcome. Conversely, intravenous injection of tPA in both wild-type and knock-out mice two hours after MCAO increased the infarct size. Furthermore, aside from excitotoxicity, additional detrimental effects stemming from tPA toxicity were identified. While detailing all of these effects exceeds the scope of this article, it suffices to mention that crucial research areas include tPA-mediated loss of BBB integrity (regulated, for instance, by PDGF-CC and MMP9 activation [29, 30] and laminin degradation [31]) and microglial activation [32]. Some effects were found to be mediated by tPA’s proteolytic activity, while others were attributed to its interaction with cell surface receptors such as N-methyl-D-aspartate receptor [33] and Low Density Lipoprotein Receptor-related Protein 1 (LRP1) [34]. These experiments underscored the dual role of tPA following ischemic stroke; beneficial in circulation as a thrombolytic, yet detrimental in the brain parenchyma as a trigger of neuronal death. Consequently, the imperative for a therapy safeguarding neurons against tPA’s toxic effects while preserving its thrombolytic activity became apparent. In this context, nervous system-specific serpins promptly emerged as promising candidates due to their potent inhibition of tPA within the parenchyma.

### Neuroserpin is a stroke-induced protein

Neuroserpin (Ns) was first isolated in 1989 in a quest to identify secreted proteins crucial for axonal growth and synapse formation [35]. Subsequent analysis of its amino acid sequence unveiled striking similarities to members of the serpin family, renowned for their role as serine protease inhibitors. Notably, examination of the sequence composition of the reactive center loop (RCL) identified Ns as an inhibitor of trypsin-like serine proteases, the best target being tPA [36, 37]. Expression studies further underscored its significance, revealing a predominant localization within neurons of the central and peripheral nervous system [37, 38], thus firmly establishing Ns as a nervous system specific serpin. Like the other members of the serpin family, Ns forms a covalent complex with its target protease, with the serine residue in the active site of the protease reacting with the P1 residue in the RCL of Ns. The RCL is thereby cleaved by the protease, thus enabling a conformational change that stabilizes the complex and inactivates the proteolytic activity of the protease [39].

The involvement of Ns in stroke was initially documented in the early 2000s. In rodent models subjected to MCAO, researchers observed an elevation in Ns levels within the ipsilateral hemisphere [40-42]. However, while Liang and colleagues reported a surge in Ns transcription as early as one-hour post-reperfusion following MCAO in rats, which persisted for the subsequent 24 hours compared to sham-operated animals [43], other research groups observed a decline in Ns immunohistochemical signal within the ipsilateral hemisphere during the initial hour’s post-ischemic stroke, followed by an upregulation in the penumbra, cortex and hippocampus [40, 42]. Likewise, increased Ns expression was noted in response to other ischemic insults. Following acute ischemic/reperfusion injury in the retina, Ns expression surged shortly after injury and remained elevated for up to 24 hours [44]. Furthermore, increased Ns expression in the cortex and hippocampus of mice was detected in response to sublethal ischemia (brief ischemic episodes with intermittent reperfusion) [45]. This mechanism, also known as ischemic tolerance [46], is an endogenous neuronal survival program involving several signaling pathways known to protect brain cells from subsequent ischemic damage. Thus, increased expression of Ns may imply a role in long-term protection. Hemorrhagic stroke in rodents also demonstrated elevated Ns levels, peaking 48 hours’ post-blood injection, coinciding with the highest behavioral deficits [47].

In stroke patients, serum Ns levels upon admission were found to be higher than those in healthy controls, subsequently experiencing a marked decrease over the following three days [48-50]. Moreover, a positive correlation was identified between Ns levels upon admission and favorable outcomes 3 months’ post-stroke, while a negative correlation was observed between the decline in Ns levels during the initial days’ post-admission and markers of inflammation, excitotoxicity and BBB disruption.

### Ns is neuroprotective in ischemic stroke

**Figure 1.**
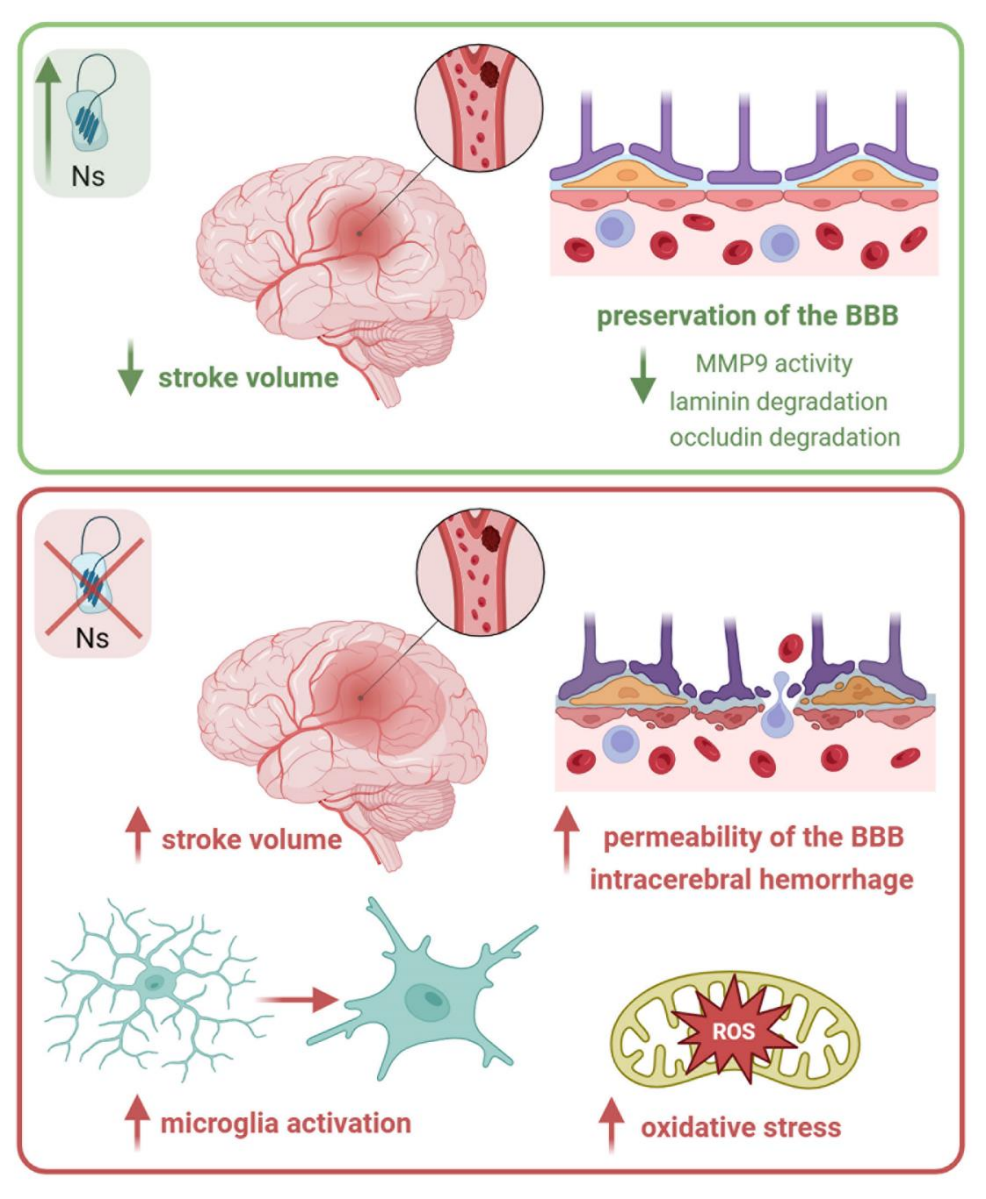
Ns is neuroprotective in ischemic stroke. Ns administration, as well as its genetic overexpression, reduce brain injury following MCAO in rodents, as evidenced by decrease in stroke volume [40, 41]. Moreover, elevated Ns levels lead to reduced MMP9 activity [30], laminin [40] and occluding degradation [51], thereby preserving the integrity of the BBB. In contrast, absence of Ns results in increased stroke volume, enhanced microglia activation [57] and loss of BBB integrity exacerbating intracerebral hemorrhage [58] in mice following MCAO. A rise in oxidative stress following hypoxic/ischemic injury has been shown in Ns-deficient zebrafish [59]. (Created with BioRender.com)

The neuroprotective role of Ns was initially demonstrated in rodents following MCAO through intracortical injection of the serpin [40] (Fig. 1). Subsequently, another study published a year later validated this observation using transgenic mice engineered to overexpress Ns in the brain [41]. In addition to reducing stroke volume, the presence of Ns mitigated the degradation of laminin, a crucial component of the basement membrane essential for maintaining the integrity of the BBB. Furthermore, Ns attenuated microglial activation and resulted in a smaller proportion of neurons undergoing apoptosis in the ischemic penumbra. Intriguingly, administration of a RCL cleaved Ns, unable to react with the target protease, failed to reduce stroke volume, highlighting the necessity of active Ns for neuroprotection [40]. Follow-up studies have further elucidated the effects of Ns on preserving BBB integrity following MCAO in mice. These studies reported a concurrent reduction in MMP9 activity and in occludin degradation as a consequence of Ns administration, underlining its protective effects [30, 51]. In the following years, researchers delved into exploring the protective capabilities of Ns across various contexts. For example, rats with chronic experimental diabetes mellitus presented an increased infarct volume following MCAO compared to their normal counterparts, correlating this heightened severity with lower levels of Ns mRNA detected in the diabetic brain [43]. In a mouse model of acute retinal ischemic/reperfusion injury, induced by elevating intraocular pressure, neuronal apoptosis led to a reduction in retinal thickness [44]. However, Ns administration prior to the lesion mitigated the number of cells undergoing apoptosis and expedited recovery, as evidenced by higher b-wave amplitudes detected by electroretinogram seven days’ post-ischemia. Explorations into Ns's neuroprotective potential extended to hemorrhagic stroke as well. In the study by Li and coworkers, injury was induced in mice via stereotactic injection of autologous blood into the basal ganglia [47]. Application of recombinant Ns directly into the hematoma at all time points studied (24, 48 and 72 hours’ post-surgery) mitigated behavioral deficits, cerebral edema and BBB permeability, as confirmed by preservation of occludin-expressing cells around capillaries. Lastly, Ns was tested as an adjuvant treatment of therapeutic hypothermia to ameliorate brain injury following perinatal asphyxia [52]. For this purpose, rats at postnatal day 7 underwent hypoxia/ischemia via ligation of the right common carotid artery and exposure to low oxygen concentration for two hours. Immediate cooling techniques were applied post-injury, either alone or in conjunction with a single intraventricular dose of Ns. As expected, hypothermia exhibited beneficial effects, and the concurrent injection of Ns further attenuated neuronal death in the hippocampus, reaffirming the neuroprotective role of the serpin.

In vitro studies have substantiated the neuroprotective ability of Ns, particularly in primary cortical neuronal cultures exposed to oxygen and glucose deprivation followed by reoxygenation (OGD/R), a well-established method for simulating ischemic damage. Notably, Ns expression increased 24 hours post-OGD/R, mirroring the post-stroke scenario in the brain [53]. Furthermore, treating primary neurons with Ns conferred a reduction in cell death, irrespective of the timing of Ns administration, 24 hours prior to, immediately preceding, or even after ODG/R [45, 53, 54]. Ns treatment effectively curbed neurite fragmentation and mitigated the proteolytic activity of MMP9 and tPA [53, 54]. Conversely, tPA administration post-OGD/R exacerbated cell toxicity, but intriguingly, concurrent Ns addition attenuated tPA-mediated neuronal loss. The neuroprotective spectrum of Ns is not confined to neurons alone; it extends to glial cell cultures as well. In primary cortical astrocytes subjected to OGD/R, Ns treatment bolstered cell viability, curtailed NO and TNF alpha secretion in the medium and inhibited the NF-kB pathway [55]. Similarly, in primary microglia cultures, Ns downregulated IL-1beta and NO production, along with dampening the mitogen-activated protein kinase (MAPK) pathway [56], suggesting a direct interconnection between Ns and neuroinflammation.

Given the neuroprotective effect associated with higher levels of Ns in the brain, it is reasonable to anticipate adverse consequences in the absence of Ns following a stroke. This premise has been evaluated across various mouse models. For example, Gelderblom et al. demonstrated that Ns-deficient mice subjected to MCAO exhibited an enlarged stroke volume, poorer neurological scores and reduced survival rates [57]. Moreover, while the penumbra area showcased a predominantly activated microglial phenotype in both Ns-deficient mice and controls, distant regions of the ipsilateral hemisphere displayed a higher incidence of amoeboid and fewer resting microglia in Ns-knockout mice compared to their wild-type littermates. In addition to exacerbating stroke lesion, the absence of Ns increased BBB leakage and intensified occurrence of spontaneous intracerebral hemorrhage [58]. These detrimental effects were ameliorated in double knockout mice deficient in both Ns and tPA, suggesting that the worsened post-stroke damage observed in Ns-deficient mice is mediated by tPA. In zebrafish models, hypoxic injury induced by cobalt chloride during embryonic stages resulted in developmental deficits such as spine deformation, pericardial edema, vascular malformations and reduced eye and brain size. These defects were accompanied by behavioral alterations and culminated in a decrease in the survival rate [59]. Ns-deficient zebrafish mutants displayed exacerbated damage, underscoring the neuroprotective role of the serpin. Notably, the disparity between Ns-deficient and control animals was discernible only following hypoxic treatment, thereby excluding that absence of Ns per se could account for developmental alterations, as observed in mice [60]. Increase in peroxidation products and the concurrent decline in the activity of antioxidant enzymes in Ns-deficient zebrafish post-hypoxia suggested that induction of oxidative stress underpinned the heightened susceptibility in the absence of Ns.

### Ns extends the time window of tPA administration

An intriguing feature of Ns is its potential to extend the therapeutic window for tPA administration. Remarkably, two independent studies showed that while early tPA administration in rats (within one hour of MCAO) reduced stroke injury, tPA injection four hours after ischemia exacerbated hemorrhagic transformation and stroke volume [42, 61]. Both investigations demonstrated that administering Ns into the cisterna magna either one hour before or concurrently with tPA reduced stroke volume, mitigated BBB injury, consequently diminishing cerebral edema, oxidative stress, and neuronal apoptosis. This finding holds significant implications, particularly in light of the above-mentioned thrombolysis limitations, as it could potentially broaden the pool of stroke patients eligible for recombinant tPA administration.

### Compartmentalization of tPA activity

As mentioned above, following ischemic stroke the activity of tPA is beneficial in the blood but harmful in the brain. This finding has been recently evidenced by a study comparing stroke outcome following MCAO in mice deficient for two tPA inhibitors: Ns, regulating tPA activity in the brain, and plasminogen activator inhibitor type-1 (PAI-1), a serpin acting as physiological inhibitor of tPA in the blood [58]. Whereas Ns-deficiency worsened stroke outcome, mice lacking PAI-1 presented smaller infarcts and increased cerebral blood flow recovery following stroke, but unchanged BBB permeability. In the same study, in an attempt to protect the brain post-stroke, the activity of tPA was differentially regulated in the two compartments, increased in the blood and reduced in the brain parenchyma. This was achieved by concomitantly administering two compounds: MDI-2268, a PAI-1 inhibitor that enhances endogenous fibrinolysis [62] and imatinib, an inhibitor of tPA-mediated PDGFRα signaling known to reduce BBB permeability in the brain [63]. The treatment decreased infarct size compared to untreated but also to single-treated mice and reinforces the idea that following stroke it might be beneficial to differentially regulate tPA activity in the two compartments: inhibition in the brain parenchyma, increase in the circulation.

### Is the inhibitory activity of Ns necessary for neuroprotection?

As described above, inhibition of the activity of a serine protease requires complex formation between the protease and the serpin. The complex is typically very stable, it interacts with cell surface receptors, is taken up and degraded by cells [39]. However, the situation is more complicated for Ns. On the one hand, kinetic assays have demonstrated Ns capability to bind to and inhibit several serine proteases, including tPA, urokinase-type plasminogen activator and plasmin, with the highest inhibitory activity observed against tPA. In the mouse brain, reduced tPA activity has been evidenced through in situ zymography in transgenic mice overexpressing Ns, while injection of adeno-associated virus expressing Ns effectively ablated tPA-mediated alterations in dendritic spine density in vivo [41, 64]. On the other hand, the complex formed between Ns and tPA in vitro is exceptionally unstable and rapidly dissociates, releasing a cleaved serpin and a fully active protease [65]. Additionally, in the brains of Ns-deficient mice, no increase in tPA activity has ever been observed [66]. The discovery that a mutant form of Ns carrying mutations in the RCL (therefore lacking inhibitory activity) can still regulate N-Cadherin-dependent cell adhesion as effectively as its wild-type counterpart has led to the hypothesis that Ns may exert its effects independently of its inhibitory activity [67].

**Figure 2.**
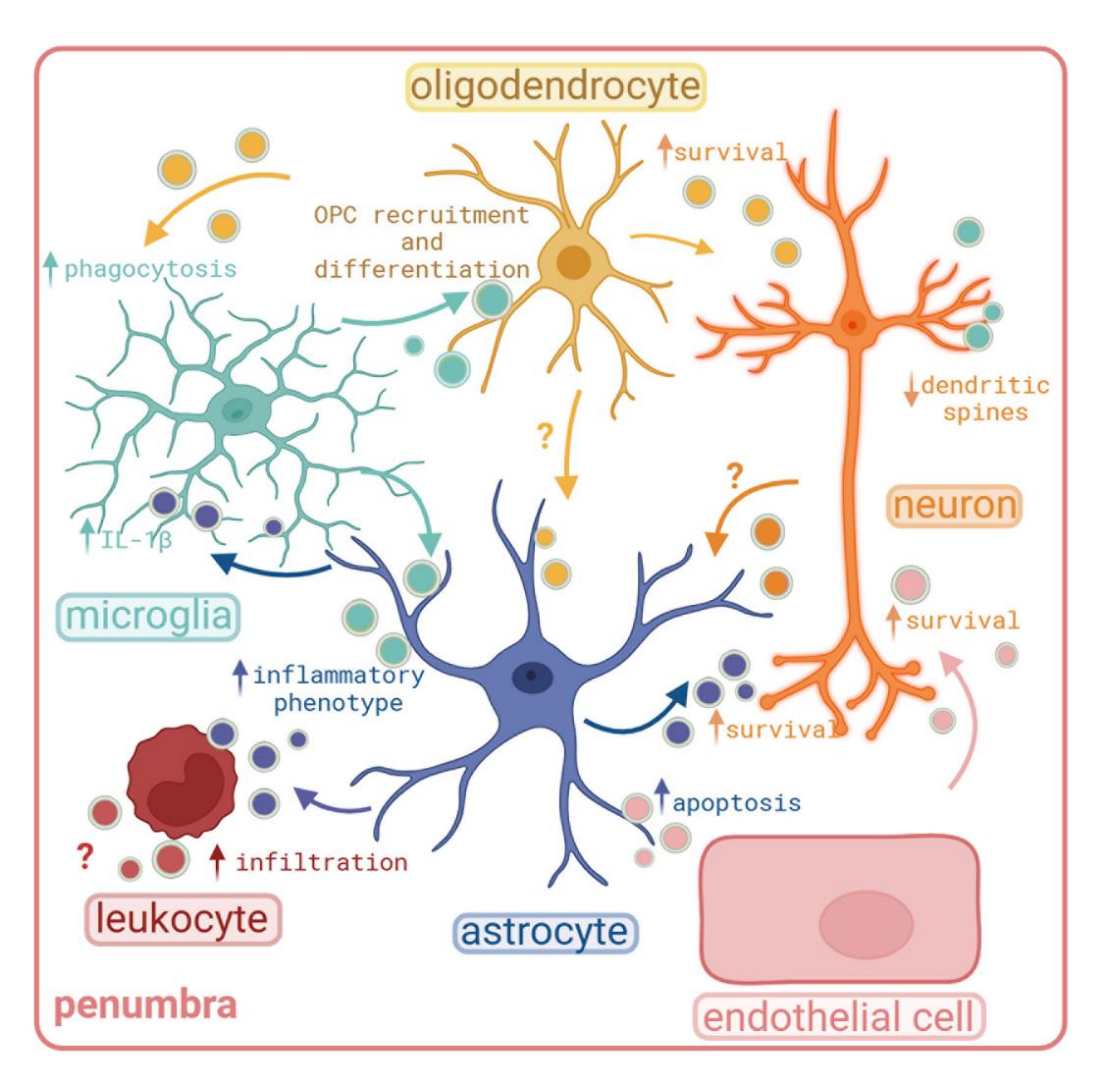
Schematic picture of EV-based communication between brain cells under injury conditions. In vitro studies have shown that after injury or inflammation, EVs released by different types of brain cells elicit different responses on the recipient cell, as explained in the main text. EVs are coloured circles (colour represents cell type of origin). (Created with BioRender.com)

The situation does not become clearer in the realm of stroke research. Plasminogen activator activity increases in the ipsilateral hemisphere following stroke, and Ns treatment, whether through intracortical or intraventricular injection of Ns or via genetic overexpression, dampens this activity [40-42, 51]. Furthermore, increased presence of Ns-tPA complexes has been detected in the hippocampus, cerebellum and neocortex of mice overexpressing Ns post-MCAO [41], reinforcing the idea that increased inhibition of tPA by Ns may underpin neuroprotection. However, Ns-induced decrease in ischemic stroke volume following MCAO and Ns-mediated recovery after ischemia in the retina have also been noted in tPA-deficient mice, hinting at a tPA-independent effect [44, 45]. Indeed, as mentioned above, a tPA-independent protection by Ns-induced preconditioning following OGD/R has been delineated in primary neurons as well. In this context, Ns preconditioning also shielded neurons from plasmin-mediated excitotoxic cells death, implying that while Ns inhibitory activity might be essential for neuroprotection, it could be directed against a protease other than tPA [45]. Interestingly, proteomic analysis has demonstrated Ns presence in extracellular vesicles (EVs) isolated from brains of Alzheimer’s disease patients [68]. Given that EVs play a role in stroke outcomes, as explored in upcoming chapters, the presence of Ns in EVs suggests an additional mechanism through which it may exert neuroprotection.

### *Roger that*: EVs messaging in ischemic damage

Once thought to be just mere trash bags where the cells disposed their unwanted products, EVs are now recognized as pivotal players in cell communication in physiological and pathological conditions [69, 70]. EVs, nanoparticle-sized entities, are universally released by all cells studied to date, spanning from protozoan parasites, bacteria, fungi, plants and insects to mammals [71, 72] and they also participate in inter-kingdom communication [73, 74]. Loaded with various cargoes such as proteins, nucleotides, metabolites and lipids that mirror the status of the cell of origin, EVs can be transferred to recipient cells, eliciting specific responses. Notably, the sorting of the cargoes into EVs is a selective process; certain miRNAs, for instance, are found in EVs and not observed in their cell of origin, where levels might be too low for detection [75, 76]. In pathological conditions such as stroke, EV content undergoes alterations [77, 78] and given their ability to traverse the BBB and be present in cerebrospinal fluid and blood, EVs emerge as very attractive biomarkers and tools to study brain pathologies, particularly since brain biopsies are seldom performed [79].

However, it is crucial to note that EVs constitute a highly heterogenous group of vesicles. Based on their origin, they fall into two categories: exosomes (derived from endocytic compartments) and ectosomes (shed from the plasma membrane). Yet, because of the diversity of cargoes and functions, further classification is warranted, encompassing small ectosomes, microvesicles, apoptotic bodies, migrasomes or exophers just to name some [80]. Consequently, as the definition and classification of EVs constantly evolve correlating with the refinement of techniques for nanoparticle-sized investigation, EV research presents formidable challenges. Not only there are still many unknowns about EV biology but also there is a need to improve reproducibility by the adoption of standardized procedures and the establishment of rigorous guidelines [81, 82].

In stroke as in other brain diseases, the interplay between astrocytes, microglia, oligodendrocytes, as well as inflammatory infiltrating cells profoundly influences neuronal fate [83]. All these cells release EVs and participate in stroke’s communication repertoire [84, 85] (Fig. 2). For instance, EVs derived from oligodendrocytes confer protection to neurons subjected to OGD/R, probably by delivering anti-oxidative enzymes such as catalase and super-oxide dismutase-1 while activating pro-survival signaling cascades [86]. Additionally, intracerebral delivery of EVs secreted by pro-regenerative microglia has been shown to promote the recruitment and differentiation of oligodendrocytes precursor cells (OPCs) in a mouse model of stroke, thereby supporting remyelination after ischemic damage [87]. As oligodendrocytes are highly vulnerable and die within hours after ischemic damage, exacerbating axonal injury [88], rescuing OPCs through EVs might be used as therapeutic treatment. Under other circumstances EVs may stimulate harmful reactions. Pro-inflammatory microglia activated by the release of ATP (as a DAMP) from dying neurons in inflammatory conditions [89], release EVs exhibiting an altered proteome compared to their homeostatic state, that triggers an inflammatory phenotype in astrocytes as demonstrated both in vitro and in vivo in a mouse model of multiple sclerosis [90, 91]. Conversely, ATP released by astrocytes induces the shedding of EVs from microglia which are loaded with IL1β, a key player in the inflammatory process [92]. Also under inflammatory conditions, pro-inflammatory microglial EVs contain miR-146a-5p, that once transferred to neurons, control the expression of synaptotagmin1 and neuroligin1, inducing dendritic spine loss in hippocampal neurons in vitro and in vivo and an overall loss of excitatory synapses [93]. In mice subjected to the permanent model of MCAO it has also been demonstrated that when TNFα signaling pathway is inhibited, pro-inflammatory microglia release less EVs, thus indicating a potential role for excitotoxicity-mediated TNFα to regulate microglia EV release [94]. Astrocytes also play a pivotal role in ischemic stroke through secretion of EVs. Thus, astrocyte-derived EVs can protect neurons subjected to ischemic conditions in vitro, a process that is dependent on the presence of the prion protein (PrP) on the vesicles [95]. Significantly, the delivery of EVs derived from primary cultures of astrocytes exposed to OGD to rat brains subjected to MCAO, results in a remarkable enhancement of axonal recovery within the affected area [96]. However, once again the contribution of EVs can also be detrimental. Astrocyte-derived EVs can cross the BBB to promote leukocyte recruitment to the brain in response to inflammation, a process mediated by IL-1β [97], exacerbating the damage [98]. Under physiological conditions, neurons facilitate the transfer of miR-132 to endothelial cells, contributing to the maintenance of vascular integrity while they transfer miR124a to astrocytes to regulate the amount of glutamate transporter GLT1 [99, 100]. Under ischemic conditions, studies have demonstrated that primary neuronal precursor cells exposed to OGD release EVs that enhance the survival and recovery of cortical neurons in vitro and, to a limited extend, to mice subjected to MCAO [101]. Finally, under ischemic conditions endothelial cells-derived EVs seems to mediate neuronal protection to neurons subjected to OGD/R and to mice subjected to transient MCAO thanks to the delivery of miR-1290 [102]. EVs released from endothelial cells also influence BBB integrity. Endothelial cells subjected to OGD/R release EVs that affect the permeability of the BBB by acting on astrocytes, a phenomenon also observed in vivo when endothelial EVs are injected into mice after transient MCAO and the leakage of the BBB as well as the overall cerebral injury is shown to be magnified [103].

Many of the aforementioned studies were conducted in vitro or by using EV populations from in vitro cultures. While these studies are essential to elucidate specific functions of EVs derived from particular cells, they may not capture the intricate temporal interplay and the complex EV composition found in vivo. In stroke, as already described above, inflammation players have a dual role, by further exacerbating damage in early phase but also needed on the recovery phase [104]. Hence, isolation or enrichment of EVs derived from tissue is also necessary to characterize the properties of EVs. In our group, we have isolated EVs from the ipsilateral hemisphere at different times after transient MCAO in mice. We observed that while microglia appear to be the primary population relatively releasing more EVs under steady-state conditions, 24 hours after stroke astrocytes release a comparatively higher quantity of EVs [105]. Furthermore, upon analyzing the mRNA content of EVs isolated from ischemic tissue at 72 hours’ post-stroke, we found several upregulated mRNAs related to stress response but also to repair processes. These mRNAs could be attributed mainly to a microglial origin [106]. Interestingly, 72 hours after MCAO oligodendrocyte EVs appear to be relatively increased. These studies highlight a temporal pattern of EV release from different cellular origin that may be crucial for understanding stroke recovery mechanisms.

### Neuroserpin in EVs?

**Figure 3.**
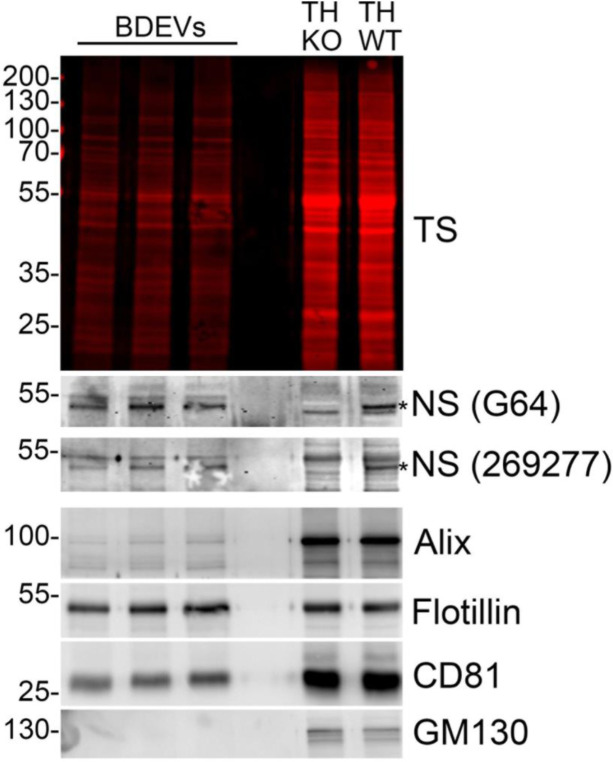
Presence of Ns in brain-derived EVs (BDEVs). Western blot analysis of three BDEV samples, isolated from the brain of wild-type mice following the protocol from Crescitelli et. al. [109]. Ns was detected in BDEVs with two different antibodies, G64 polyclonal antibody [120] and #269277 (1:1,000; abcam). Correct identity of the BDEVs is demonstrated by antibodies against EV markers Flotilin-1 (1:1,000; #610820; BD Biosciences), CD81 (1:1,000; #10037; Cell Signaling Technology), and Alix (1:1,000; #12422-1-AP; Proteintech). As expected, the Golgi marker GM130 (1:1,000; #61082; BD Biosciences) is absent from the BDEV-samples, indicating no contamination with intracellular organelles. Total protein staining (TS) was performed using the Revert Total Protein Stain Kit (LI-COR) to ensure similar protein loading. As a control for Ns, hippocampal extracts from adult Ns-deficient mice and wild-type littermate (TH KO and TH WT, respectively) are used, Ns (asterisk) is present in the wild-type but not in the knockout sample. EV size (mean: 192.1 ±3.1 nm; mode: 143.77 ±5.3 nm) and concentration (1.28e+012 ±3.19e+010 particles/ml) were measured by NTA 3.0 software (Nanosight, Malvern). Data in the figure are unpublished original work.

As a secreted protein, Ns is not expected to be found in EVs. However, its presence has been described in EVs isolated from human urine [107] and brain [68]. Additionally, it is not the sole member of the serpin protein family identified in EVs: a mutant form of α1-antitrypsin that accumulates in the endoplasmic reticulum (ER) has been detected in EVs, probably for disposal [108] and we have found the wild-type α1-antitrypsin to be present in our proteomic analysis of brain-derived EVs from wild-type mice [105]. We have now isolated EVs from murine brain following the protocol of Crescitelli et al. [109] and we confirmed the presence of Ns within these EVs, as depicted in Figure 3 (unpublished results). As other secreted proteins, such as TGFβ1, are present in EVs, there must be a mechanism which loads these proteins, which otherwise follow the secretory pathway, into these vesicles. One plausible explanation is that they become part of the EV corona, a protein coating that forms around the vesicles upon their release into the extracellular space and formed by proteins that are present already in the extracellular matrix (i.e. they are released as secretory proteins and then recruited to the surface of EVs) [110]. These proteins, despite not being enclosed inside the EVs themselves, play important functions in EV uptake by recipient cells and in immune-recognition [111, 112]. Alternatively, it is conceivable that certain secretory proteins are indeed encapsulated within EVs. Recent findings have highlighted the role of the ER membrane contact sites (ER MCS) with endosomes in the regulation of the biosynthesis of a subset of exosomes enriched with RNA. This process facilitates the incorporation of ribonucleotide proteins and RNA to EVs [113], suggesting the existence of a biosynthetic EV pathway connected to the ER. A certain degree of exchange between ER and endosomes, without fusion events between these two organelles (the distance in the MCSs between ER and endosomes is maintained at around 3-15nm) has been confirmed by other studies. Lipids such as phospholipids and sterols, synthesized at the ER, can be transferred to endosomes at their contact points with the ER [114]. The epidermal growth factor receptor is dephosphorylated by PTP1, a phosphatase that is present at the cytosolic face of the ER. This process occurs at the ER-endosome MCSs using LRRK1 as a scaffolding protein, and it implies a certain degree of exchange between compartments [115]. Within this context, proteins destined for secretion could potentially be loaded in EVs during their biogenesis. Indeed, proteins related to the extracellular matrix such as Tenascin C have been recently shown to be transferred from the ER to multivesicular bodies, a regulated sorting that involved ER-endosomal MCSs and caveolin-1 [116]. Further studies are thus warranted to not only pinpoint the mechanism leading to Ns internalization in EVs but also its potential physiological function(s).

### Conclusions

Several agents have been evaluated for therapeutic purposes in acute ischemic stroke, though clinical set-ups have not yet been achieved [117]. The lack of clinical translation might be explained by different reasons, one of them being the challenge of a successful drug delivery to the brain, bypassing the BBB [118]. Another significant hurdle in stroke treatment is the dual nature of its molecular processes: what may be detrimental in the early stages of the disease can become beneficial during the later recovery phase and vice versa, complicating efforts to target these dynamic mechanisms as the condition progresses [1].

Ns treatment after ischemic stroke has demonstrated to be beneficial both in vitro and in vivo. Ns overexpression in mice is well tolerated: abnormal emotional behavior has been observed when overexpression starts during brain development, but not in the adult hippocampus [66, 119], making time-limited administration following stroke conceivable. Future challenges to allow translation into a clinical application include, besides brain delivery, the investigation of long-term outcome, as consequences of Ns treatment have only been assessed a few days after MCAO in rodents. Additionally, determining the optimal timing and frequency of Ns administration will be crucial. Moreover, understanding the mechanisms behind Ns neuroprotective role might allow to better modulate its administration. It remains to be clarified whether the inhibitory function of the serpin is required, and the pathways involved in neuroprotection need characterization. In this regard, the discovery of Ns presence in EVs offers new perspectives, as it might provide insights into the functions of the serpin in the interplay and communication between distinct brain cell types, thus advancing our understanding of the mechanisms underlying neuronal recovery within the penumbra.
